# Predictors of emergency contraceptive use among regular female students at Adama University, Central Ethiopia

**Published:** 2010-11-26

**Authors:** Faten Dejene Tilahun, Tsion Assefa, Tefera Belachew

**Affiliations:** 1 Department of Health Promotion and Behavioral Sciences Jimma University, Ethiopia; 2 Department of population and Family Health, Jimma University, Ethiopia

**Keywords:** Emergency contraception, contraception, predictor, Adama University, Ethiopia

## Abstract

**Background:**

One of the key interventions to reduce unintended pregnancy and unsafe abortion outlined in the national youth strategy is availability of emergency contraception. However, there are no studies which document emergency contraception use and the factors influencing the use of emergency contraceptives among university girls in Ethiopia. This study was carried out to assess emergency contraception use and its predictor factors among regular female students at Adama University.

**Methods:**

A cross-sectional study was conducted during the month of February 2009, among randomly selected 660 female students of Adama University Central Ethiopia. Data were collected through pre-tested selfadministered questionnaire. Bivariate and multivariable logistic regression models were fitted to identify variables predicting emergency contraception use.

**Results:**

One hundred ninety four (29.4%) students were sexually active and 63 (9.4%) had a previous history of pregnancy. And most of the pregnancies (92%) were unintended. Majority (77.7%) of pregnancies were terminated by way of induced abortions carried out by untrained persons. Only 26.7% of those who had unprotected sex used emergency contraception. Lack of knowledge, fear of being seen by others, and inconvenient service delivery were pointed out as the main reasons for not using emergency contraceptives. Previous use of contraceptives (AOR: 1.953; 95% CI = 1.72- 6.345), being married (AOR: 9.254; 95% CI = 2.538-20.73) and age of 20 years and above (AOR: 2.372; 95% CI = 1.102-7.246) were significant predictors use of emergency contraception, while poor knowledge of emergency contraception was a significant predictor of non-use of emergency contraception (AOR: 0.09; 95% CI = 0.041-0.189).

**Conclusion:**

The study pointed out the need for increasing the knowledge of university going young women about emergency contraception, and the need for availing youth friendly reproductive health services to promote preventive behavior.

## Background

Despite the technological advancements in modern contraception methods, unintended pregnancy is still a big problem in Ethiopia [1-2]. More than 60% of the pregnancies in adolescents are unintended; ones which result from contraception non-use, contraception method failure and rape [3-4]. The incidence of unintended pregnancy and unsafe abortion, particularly among adolescents, remains high [1,5,6]. In Ethiopia, abortion emanating from unintended pregnancy is one of the most significant causes of maternal morbidity and mortality; it is also a major medical and public health problem [1,2].

Currently, more and more young people engage in sexual activity before marriage, often without using contraception [1]. Country-specific data indicates that young women who are unmarried are increasingly sexually active before the age of 15 [7,9]. Thus unwanted pregnancy is one of the greatest problems a young girl can face; this poses major public health problems in the developed and developing countries, including Ethiopia [11, 12]. Unintended pregnancy and early child bearing impacts negatively on the educational prospects of girls by forcing them to drop out of school (jeopardizing students' educational progress and future careers) because of the morbidity resulting from unsafe abortion when the pregnancy is unwanted, culminating in poor participation of girls in the overall socio-economic development of their communities and eventually their countries [6,7].

The use of emergency contraception (EC) will decrease the cost, the emotional and the physical risk experienced by women of reproductive age who engage in early sexual activity [3,7]. EC refers to the type of contraception that is used as an emergency procedure to prevent unintended pregnancy following an unprotected act of sexual intercourse or contraception failure [7].

There are four forms of EC methods used as emergency contraceptives: Levonorgestrel (LNG)-only regimen. 0.75 mg LNG (or 1.5 mg norgestrel) taken as soon as possible after unprotected sex but optimally within 72 hours. This dose should be taken a second time, 12 hours after the first dose or a 1.5 mg single levonorgestrel dose can substitute two 0.75 mg doses 12 h apart up to 120 hours after unprotected intercourse; Yuzpe combined oral contraceptives (COC) regimen: 100 mcg ethinyl estradiol plus 0.5 mg of LNG (or 1.0 mg norgestrel) taken as soon as possible after unprotected sex but optimally within 72 hours. This same dose should be taken a second time, 12 hours after the first dose. A single 10 mg dose of mifepristone are effective for emergency contraception up to 120 hours after unprotected intercourse and Ulipristalacetat 30 mg and IUD insertion taken as soon as possible after unprotected sex but optimally within 120 hours (within 5 days) [3,7,23].

In Ethiopia the need for emergency contraception (EC) was identified in the late nineteen nineties. In 2001, the Family Guidance Association of Ethiopia (FGAE) in collaboration with the Population Council initiated EC in selected youth center clinics in the country and study area. In this project, EC was provided in a repackaged brand so it would appeal to adolescents and youth in several ways for example, by cutting the cost of regular contraceptive pill, though the services were limited in scope and coverage [24]. However, after the survey, the availability and accessibility of EC were ensured in both public and private sectors of the country as well as in the study area. The following are the emergency contraceptives that are currently in use in the study area: (1) Combined Oral Contraceptive Pills (COCPs): an increased dose of combined oral contraceptives containing ethinyl estradiol and levonorgestrel (Yuzpe's regimen); (2) Progesterone Only Pills (POPs): high dose Progesterone Only Pills containing levonorgestrel; (3) Intrauterine Contraceptive Devices (Copper Releasing Intrauterine Contraceptive Devices) and (4) Mefipristone (Ru486): anti progesterone [24].

Reports from developed countries show that the use of EC varies from place to place [15] and the knowledge on correct use varies from 83% in Sweden [13] to less than 60% in developing countries [9]. One of the lowest percentages (10%) was observed in a study done in Ethiopia at the Addis Ababa University and Unity University College, Ethiopia [4] on the knowledge, attitudes, and practices affecting the use of EC. Findings from several studies indicate that even women, who indicate that they know how to use EC, often report they have never used it. [7,8,13,15,16].

However, there are few studies which document the extent of emergency contraception use and the influencing factors on its use among university girls in Ethiopia. This study was carried out to assess EC use and its predictor factors among regular female students at Adama Page number not for citation purposes 3 University. We hope that our study will provide baseline data to assist policy makers in developing appropriate evidence-based strategies to promote the need based use of emergency contraceptive methods amongst eligible individuals in Ethiopia.

## Methods

This study was conducted in Adama University, located 100 Km south east of Addis Abbaba. According to the information obtained from the Registrar Office of Adama University during the study period, Adama University had a total of 11788 students during the academic year of 2008 - 2009 of which 3,206 (27.2%) were females. The University is located in Adama town where there is a busy transportation center. The city is situated along the road that connects Addis Ababa with Dire Dawa. A large number of trucks use this route to travel to and from the seaport of Djibouti. Additionally, the Addis Ababa-Djibouti railroad runs through Adama. The number of hotels, restaurants, pubs and clubs are increasing from time to time and in much the same way, the numbers of commercial sex workers and their clients are greater than before.

A cross-sectional study was conducted during the month of February 2009, using all regular undergraduate female students of Adama University as a source and randomly selected students as study participants.

The sample size was determined using a single population proportion formula assuming; 95% level of confidence proportion of EC use of 73.4 % [19], a design effect of 2 and non-response of 10%. This gave a final sample size 660 female students. A two-stage sampling approach was used; where first 25 departments were selected randomly from the total 45 departments of Adama University (from four schools namely, School Of Business Administration, Management and Trade, School of Engineering and information Technologies, School of Humanities and Natural Science, School of Pedagogic and Vocational Teacher Education). Then, the total sample size was allocated to each department proportional to the number of female students per department. Secondly, from each respective department, participant students were selected proportional to their year of study using simple random sampling technique. The stratification of the year of the students was because the variable of interest in the study may vary across the year. Finally, 660 study participants who fulfilled the inclusion criteria were selected for the study.

The questionnaires contain five parts namely; socio-demographic characteristics, sexual and reproductive issues, practice of regular contraceptive use, knowledge EC, attitude of EC and utilization of EC.

The first part assessed information on the socio-demographic characteristics of the study participants which consisted of 9 questions. The second part assessed the sexual and reproductive issue and contained 10 questions. The questions were asked in the form requiring “Yes”, “No” response and in multiple response form. Some of the questions were: ( 1) Ever had sexual experience? (2) Have you ever been pregnant? (3) What was the main reason for unwanted pregnancy? (4) Have you ever experienced induced abortion? All this questions were close ended (Yes, No and/or multiple choice questions). The third part contained practice of regular contraceptive use and knowledge of EC and consisted of 9 questions. The fourth part contained their attitudes and consisted of 6 questions. The fifth part assessed their prior EC practice and consisted of 6 questions.

Knowledge of EC was assessed by asking 8 questions. The eight questions evaluating the level of knowledge about EC were: (1) Which of these is an emergency contraceptive pill?, (2) How frequently EC can be used after unprotected sexual intercourse?, (3) Is EC is a method of early abortion?, (4) When taken early, can EC prevent sexually transmitted infections?, (5) Where can emergency contraception can be obtained? (6) When will emergency contraceptive pills be effective?, (7) When can IUCD be effective as an emergency contraceptive?,(8) How effective is it to use EC Pills in preventing pregnancy? . Each question was corresponds with “Yes” or “No” response and all “Yes” responses were given a value of “1” while “No” responses were given a value of “0”. The cut-off value used was taken from the mean of the sampled population which is 3.3 ± 0.42. Girls who scored the mean and above were labeled as having “good knowledge (knowledgeable)” while those who scored below the mean were labeled as having “poor knowledge” (inadequate knowledge).

Students' attitudes were measured using six items rated on a four-point Likert scale as (1) strongly disagree, (2) disagree, (3) agree and (4) strongly agree. The four items were: (1) EC causes a loss of confidence between regular partners, (2) It is a good idea to avail EC for all females., (c) The service in campus or nearby clinics is convenient to use EC (4) It is good to use EC after unsafe sexual intercourse (5) It is sinful to apply EC methods "(6) EC use may cause infertility in a woman. Attitude score was computed using the above six constructs whose theoretical value ranges from 6 to 24. This scoring was subsequently reversed for negatively stated statements. Subsequently, respondents who scored above the average value of attitude score of the sampled population which is 14 ± 1.8 were labeled as having a positive attitude and respondents with an attitude score of less than the average were labeled as having a negative attitude.

The fifth part of the instrument required the students to state their prior experience with EC with either a “Yes' or “No” response and with multiple responses. It consisted of 10 structured questions. Some of the questions were: (1) Have you ever sexually active? (2) Have you ever engaged in unprotected sex? (3) Have you ever used emergency contraceptives? (4) Which methods have you used as EC? (5) In what time have you taken the method? The total numbers of question were 40 and the questions were adapted and modified according to the local situation from similar studies. The questions were either closed with “Yes”, “No” responses or involved multiple responses.

The data was collected using a pre-tested structured self-administered questionnaire which was adapted from similar survey used by similar studies [3,4,7]. The questionnaire was prepared in English and translated into Amharic and then retranslated back to English to check its consistency. Internal consistency reliability estimate of cronbach's alpha was computed for the questionnaires of knowledge and attitudes towards EC after pretest and a value superior or equal to 0.7 was considered as reliable [25]. The cronbach's alpha coefficient of knowledge and attitude were 0.86 and 0.79 respectively.

Data were cleaned, checked for inconsistencies and missed values, coded and entered for analysis to SPSS (SPSS Inc. version 16.1., Chicago, Illinois). Bivariate analysis was used to see the unadjusted effects of each predictor. Variables that showed significant association in the bivariate analyses were fitted in to a multivariable logistic regression model to isolate the independent effects on EC use. All test were two sided and statistical significance was set at p<0.05.

Ethical permission was obtained from Adama University to conduct the study. All the study participants were informed about the purpose of the study and written consent was secured from all participants prior to the start of data collection. Privacy and confidentiality of information given by each respondent was maintained and names given were not recorded. With the help of assistants from each school, the selected students were taken to one hall, where they were informed about the purpose of the study, the importance of their participation and verbal consent was obtained. Based on their willingness to participate in the study, they were provided with the questionnaire and oriented on how to fill the questions. After they had completed filling in the questionnaire they, each, returned it to the facilitator (i.e. the respondents returned their questionnaires in person).

All filled questionnaires were checked for completeness, accuracy, clarity and consistency by the facilitator and investigator. Necessary corrections and changes were made in time. All supervision by the principal investigator throughout the data collection was carried out. This was to help identify problems that had to be addressed both on the questionnaires and with the data collectors.

## Results

A total of 660 students were involved in the study. The mean (±) age was 20.2 (± 1.7) years. Majority of the students (70.9%) were Orthodox Christians, followed by Protestants and Muslims accounting for 14.4% and 13.0%, respectively. Majority (92.4%) of the respondents were living within the campus. Nearly half of (47.7%) respondents were in their first year while the rest were second (37.4%) and third year (14.8%) students (Table 1).

Nearly one third of, 194 (29.4%) respondents were ever sexually active, and out of which 37(19.1%) started sexual activity before the age 15 years and 144(74.2%) started sexual activity between 15 and 19 years of age.

Sixty three (32.5%) of the previously sexually active girls and 9.5% of all girls had been pregnant at least once. Majority (69.8%) of the pregnancies occurred between 15and 19 years of age and most of them (92%) were unintended. Forgetting to take contraceptives (41.4%), rape (18.96%) and contraception failure (13.8%) were the main reasons for unintended pregnancies .The majority (84.5%) of pregnancies culminated in induced abortion; the reasons for the termination of the pregnancy were fear of interrupting schooling (67.3%) and fear of family and community (32.7%). A considerable proportion of induced abortions were performed by untrained persons (55.1%) and some (30.6%) were by trained personnel at clinics (Table 2).

**Figure 1:**
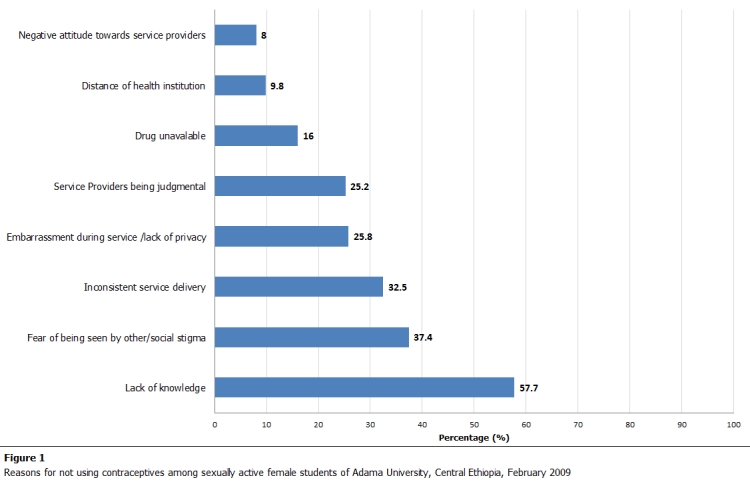
Reasons for not using contraceptives among sexually active students of Adama University

Among the girls who had ever been sexually active only 16 % used EC and from those girls who had unprotected sex only 26.7% used EC. EC and pills were the most common methods used (74.2%). However, only 35.5% used EC within the recommended time limit (that means within 72 hrs for ECPs and 5 days for IUCD) while the remaining 64.5% did not (Table 3). The most significant individuals influencing the use of EC were stated as: female friends (54.3%), boy friends (41.9%) and health workers (35.5%). Lack of knowledge (57.7%), fear (37.4%), and inconvenient service delivery (32.5%) were mentioned as main reasons for not using EC (Figure 1).

On bivariate analyses, age, year of study in campus, being married, religion, history of pregnancy, age at first sexual intercourse, previous use of contraceptives, knowledge about EC and attitude to wards EC had statistically significant association with EC use (P<0.05).

Accordingly, those who had age 20 years and above were 3.48 times more likely use EC compared to younger (COR:3.48; 95% CI = 1.802-10.10). Moreover, as year of study in campus increased, there was a relative increase in the use of emergency contraceptives COR= 3.25 (95% CI = 1.327, 7.972) for year two, and COR=3.385 (95% CI = 1.57-9.901) for year three students. Participants who were married were 15.39 times more likely to use EC compared than their counterparts (COR: 15.39; 95% CI = 7.14-33.19). Similarly female students who started sexual intercourse at late age; that means age 20 and above, were found to be 2.368 times more likely to practice EC than their counter parts who started sexual activity earlier COR= 2.368 (95% CI = 1.598, 4.105). Besides, emergency contraceptive use was higher among the Protestants compared to Orthodox and Muslim religions (COR= 4.05; 95% CI = 1.762, 9.32)

Respondents who had a history of pregnancy were found 4.9 times more likely to utilize EC than their counterparts COR= 5.68 (95% CI = 2.48, 13.007) (Table 4). And emergency contraceptive utilization was significantly higher among the respondents who had previously used regular contraceptives than those who had no experience of regular contraceptive use COR= 3.28 (95% CI = 1.49, 7.206).

Emergency contraceptive use was significantly higher among the respondents who had a positive attitude to EC than those who had negative attitude COR= 5.867(95%CI: 1.764, 19.51). On the contrary, those who have poor (lacked) knowledge about EC were 99% less likely to use EC (COR: 0.01; 95% CI = 0.057-0.209) (Table 4).

On the multivariable logistic model after adjusting for other covariates, those who had age 20 years and above were 2.4 times more likely use EC compared to those younger (AOR:2.372; 95% CI = 1.102-7.246). Similarly, those who were married were 9.3 times more likely to use EC compared than their counterparts (AOR: 9.25; 95% CI = 2.538-20.73). On the contrary, those who have lacked knowledge about EC were 90% less likely to use EC (AOR: 0.09; 95% CI = 0.021-0.189).Respondents who had experience of regular contraceptive use were 1.95 times more likely to use EC than other counterparts (AOR: 1.953; 95% CI = 1.72- 6.345) (Table 4).

## Discussion

Our results show that utilization of EC was low; correct use was even lower. The age of the respondents, marital status, and knowledge about EC and previous use of regular contraceptives as variables were found to be major predictors of EC utilization.

In this study it was found that 26.7% of those who had unprotected sex used EC, which is similar to the report of studies among university students in Addis Ababa and Jamaica [4,21] and a relatively higher proportion of EC practice was reported from South Africa and Nigeria [7,8]. The possible reasons for a low EC practice observed in this study might be related to the lower proportion of sexually active students in Adama university (29%); compared to (57%) at the university in South Africa and (63%) at university in Nigeria. It might also be due to a lack of knowledge of EC observed in this study.

Girls who were older were found to have used EC more than their younger counterparts. This finding is consistent with the study conducted in AAU, Ethiopia, South Africa, Nigeria and France, which reported that age has a significant effect on the practice of EC, where older age groups are more likely to use EC when compared to younger age groups [4,7-9]. Younger girls may have less information about the proper use of EC due to the fact that they are newly enrolled in university and may not have received this information in prior schooling

In the current study, marital status showed a significant association with use of EC. This finding is comparable to the reports of studies conducted in Addis Ababa University [3] and among women of reproductive age groups in Mongolia which showed that married female students were more likely to practice EC than unmarried girls [9]. The effect of marital status and increment in age on EC use might be linked to issues like decreased fear of being seen by others for those older and married girls. In addition, better exposure to information, maturity and heightened awareness of the consequences of unintended pregnancy held by girls as they get older and engaged in marital status that means older/married women would have less severe consequences from unintended pregnancy than would their younger unmarried counterparts [4,13,14,18].

In this study, good knowledge of EC was a significant predictor of their use, which is in agreement with reports of studies conducted in Nigeria [8], Cameroon [13] and Sweden [14] where knowledge of EC was significantly associated with increased likelihood of using them. The study also showed that experience of using regular contraception had a significant association with EC use, where those who used regular contraceptive methods used EC more compared to those who had no previous experience of regular contraceptive use. This finding is inconsistent with the reports of other studies which showed that a lower proportion of girls with experience of regular contraceptive use used EC [9,14]. This inconsistency might be explained by the differences in level of use of regular contraceptives in the studies which were not further explored.

Although the findings of this study may not be generalized to girls who are out of university, it has demonstrated the sexual and reproductive health problems faced by girls in Adama University. However, the findings may not be representative of all higher learning institutions of Ethiopia as the socio-cultural situations around the different Universities in Ethiopia vary greatly. Although the anonymous self-administered questionnaire was used, the possibility of social desirability bias cannot be totally eliminated as the study touches sensitive issues. In general this study came up with findings which have a policy implication of reducing the short and long term effects of unintended pregnancy among young girls in higher learning institutions. The need for increasing the knowledge of university girls about EC and availing youth friendly sexual and reproductive health services is implicated.

## Conclusion

In general this study came up with findings which have a policy implication of reducing the short and long term effects of unintended pregnancy among young girls in higher learning institutions. The need for increasing the knowledge of university girls about EC and availing youth friendly sexual and reproductive health services is implicated.

## Competing interests

The authors declare they have no competing interests.

## Authors' contribution

ICMJE authorship criteria are met. Dejene T: Concept and design, data collection and analysis, interpretation of data, initial draft, review of literature and final write up. Tsion A: Critical review of initial draft and article and final write up. Tefera B: Critical review of draft and article and final write up.

## Acknowledgments

Jimma University is highly acknowledged for financial support. And we are very grateful to regular female students at Adama University for sacrificing their time and for actively participating in this study.

## Figures and Tables

**Table 1:** Socio-demographic characteristics of female students, Adama University, Central Ethiopia, February, 2009

**Socio demographic characteristics n=660)**	**Number**	**Percent (%)**
**Age**		
15-19	220	33.3
20-24	427	64.7
25-29	7	1.1
>30	6	0.9
**Year of study**		
Year I	315	47.7
Year II	247	37.5
Year III	98	14.8
**Residence**		
Campus	610	92.4
Out of Campus	50	7.6
**Religion**		
Orthodox	468	70.9
Muslim	86	13.0
Protestant	95	14.4
Catholic	7	1.1
Others	4	0.6
**Ethnicity**		
Amhara	259	39.2
Oromo	243	36.8
Tigrie	52	7.9
Guragie	61	9.2
woliata	41	6.4
Others	4	0.5
**Marital status**		
Single	597	90.5
Married	61	9.2
Divorced	2	0.3
**Number of children**		
None	629	95.3
One	24	3.6
Two	7	1.1

**Table 2:** Reproductive history of female Adama University students, South Eastern Ethiopia, February, 2009

**Variable**	**Frequency**	**Percent (%)**
**Sexually Active (n= 660)**		
Yes	194	29.4
No	466	70.6
**Unprotected sexual intercourse(n= 194)**		
Yes	116	59.8
No	78	40.2
**Age of first sexual intercourse (n = 194)**		
Younger than 15 years	37	19.1
15-19 years	144	74.2
20+ years	13	6.7
**Ever been pregnant (n=194)**		
Yes	63	32.5
No	131	67.5
**Age at first pregnancy (n= 63)**		
Younger than 15 years	18	28.6
15-19 years	44	69.8
20+ years	1	1.6
**Unintended pregnancy (n=63)**		
Yes	58	92.1
No	5	7.9
**Reasons for unintended pregnancy (n=58)**		
Forget to take contraceptive	24	41.4
Rape(Forced to have sex)	11	18.96
Contraceptive failure	8	13.8
Rapture of condom	7	12.0
Lack of knowledge about EC	5	8.6
Abandoned (pressure) by partner	3	5.2
**Experience of induced abortion (n=58)**		
Yes	49	84.5
No	7	12.1
I don't know	2	3.4
**Place of induced abortion (n=49)**		
Untrained abortionist	27	55.1
Clinics	15	30.6
Self-infliction	7	14.3
Reasons for having induced abortion(n=49)*		
Reasons for having induced abortion(n=49)*	33	67.3
Fear of parents and family	16	32.7
Economic problems	6	12.2

*Multiple responses are possible. Sexually Active is taken to mean either previously or currently sexually active. Unprotected sexual intercourse is taken to mean either with a previous or current history of engaging in unprotected sexual intercourse

**Table 3:** Emergency contraceptive Practice among female students of Adama University, Adama, Central Ethiopia, February, 2009

**Characteristics**	**Number**	**Percent (%)**
**Ever Used EC among sexual active(n= 194)**		
Yes	31	16
No	163	84
**Ever Used EC among unprotected sex(n= 116)**		
Yes	31	26.7
No	85	73.3
**Methods used as EC (n=31)**		
EC pills	23	74.2
IUCD	2	6.4
I don't know/remember	6	19.4
Place obtained (n=31)*		
Pharmacy	15	48.4
Government health institutions	12	38.7
Private clinics	4	12.9
**Time EC were used (n=31)**		
Correct(with in 72 hrs for ECPs & 120hrs for IUCD)	11	35.5
Do not(out of the 72 hrs ECPs & 120 for IUCD)	20	64.5
**How many times used (n=31)**		
Once	15	48.4
Two and above	6	19.4
Not remember	8	25.8
I don't know	2	6.4
Told to use EC (significant others) (n=31) *		
Friends female/peers	17	54.3
Boyfriends/partner	13	41.9
Health worker	11	35.5
Teachers in the class	2	6.5

Ever Used EC (n= 194) means those that used ever EC from previously sexually active girls and Ever Used EC (n= 116) means those who have ever used EC from those girls who had ever engaged in unprotected sexual intercourse

**Table 4:** Predictors of emergency contraceptive use among Adama University female students, Central Ethiopia. February, 2009

**Predictors**	**Used Contraoceptive Yes**	**Emergency No**	**COR(95% CI)**	**AOR(95% CI)**
**Age**				
15-19 years	6(2.7)	214(97.3)	1.00	1.00
20+ and above	25(5.7)	415(94.3)	3.481(1.802,10.10) 2	2.372(1.102-7.246)^1^
**Year of study**				
year one	7(2.2)	308(97.8)	1.00	1.00
year two	17(6.9)	230(93.1)	3.252(1.327-7.972)^1^	3.147(0.944-10.485)
year three	7(7.1)	91(92.9)	3.385(1.157-9.901)^1^	3.879(0.905-16.637)
**Religion**				
Orthodox	13(2.8)	455(97.2)	1.00	1.00
Muslim	7(8.1)	79(91.9)	1.31(0.404-3.53)^1^	0.176(0.080-1.86)
Protestant/Catholic	11(10.4)	95(89.6)	4.05(1.762-9.32)^1^	1.189(0.762-5.417)
**Marital status**				
Never married/single	14(2.2)	583(97.7)	1.00	1.00
Married	17(29.0)	46(73.0)	15.39(7.14-33.19)^3^	9.254(2.538-20.73)^3^
**Number of children**				
None	23(3.7)	606(96.3)	1.00	1.00
One and above	8(25.8)	23(74.2)	9.164(3.704-22.67)	4.0811(0.989-13.82)
**Age of first sexual intercourse**				
Younger than 15 years	3(8.1)	34(91.9)	1.00	1.00
15-19 years	21(14.6)	123(85.4)	1.657(1.23,4.49)	1.720(0.946,2.959)
20+ years	7(53.8)	6(46.2)	2.368(1.598,4.105)^1^	2.38(0.869,1.228)
**History of pregnancy**				
yes	21(32.3)	44(67.7)	1.00	1.00
No	10(7.8)	119(92.2)	5.680(2.480-13.007)^1^	3.32(0.780-12.007
**Ever used RC**				
yes	13(10.1)	114(89.9)	1.00	1.00
yes	18(26.9)	49(73.1)	3.278(1.49-7.206))	1.953(1.72-6.345) )
**Knowledge of EC**				
Good knowledge	23(29.1)	54(71.1)	1.00	1.00
Poor knowledge	8(3.5)	223(96.5)	0.10(0.057-0.209) )	0.09(0.041-0.189))
**Attitude**				
Negative	6(2.4)	240(97.6)	1.00	1.00
Positive	25(6.8)	389(93.2)	5.867(1.764,19.51) )	2.567(0.564,6.43)

RC: Regular contraceptive, EC: Emergency contraceptive, 1 P< 0.05, 2 P<0.01, 3 P<0.001, COR= Crude odds Ratio, AOR= Adjusted odds ratio
